# RACIAL DIFFERENCES IN PHYSICAL ACTIVITY IN NURSING HOME RESIDENTS WITH COGNITIVE IMPAIRMENT

**DOI:** 10.1093/geroni/igz038.599

**Published:** 2019-11-08

**Authors:** Nicole A Viviano, Elizabeth Galik, Barbara Resnick

**Affiliations:** 1 University of Maryland, Baltimore, Baltimore, Maryland, United States; 2 University of Maryland School of Nursing, Baltimore, Maryland, United States

## Abstract

INTRODUCTION: Nursing home residents with moderate to severe cognitive impairment are mostly sedentary. It is more likely that African-American (AA) older adults tend to be more sedentary than their white counterparts. The purpose of this study was to examine racial differences in overall time spent in physical activity (PA), time in sedentary, light intensity, and moderate levels of PA, and participation in activities of daily living (ADLs) among cognitively impaired nursing home residents. METHODS: This was a secondary data analysis from the Function and Behavior Focused Care Intervention study. The sample included 336 cognitively impaired residents from 12 nursing homes. RESULTS: The mean age of the residents was 86.2 (SD=10.1) with an average MMSE score of 7.8 (SD=5.0) where 41% were AA and 59% white. White and AA participants engaged in only 51.2 and 46.1 minutes of light and 1.5 and 1.1 minutes of moderate level PA, respectively. There was a significant difference in time spent in light-intensity PA with whites spending more time in this level of activity [F(4, 242) = 3.360, p = .01]. Conversely, AAs had better functional ability than white residents [F(4, 242) = 4.754, p < .001]. There were no significant racial differences in time in sedentary, or moderate level PA. DISCUSSION: These findings are consistent with prior research showing that AAs had lower PA levels compared to their white counterparts. Future research should focus on increasing PA among nursing home residents and consider specific interventions to increase activity among AA residents.

